# What textbooks offer and what teachers teach: an analysis of the Dutch reading comprehension curriculum

**DOI:** 10.1007/s11145-021-10244-4

**Published:** 2022-01-06

**Authors:** Suzanne T. M. Bogaerds-Hazenberg, Jacqueline Evers-Vermeul, Huub van den Bergh

**Affiliations:** Utrecht Institute of Linguistics OTS, Utrecht University, Trans 10, 3512 JK Utrecht, The Netherlands

**Keywords:** Reading comprehension instruction, Materials analysis, Enacted curriculum, Mixed methods, Reading strategy, Text structure

## Abstract

In the Netherlands, the quality of the reading curriculum is currently under debate because of disappointing results on national and international assessments of students’ reading skills and motivation. In a mixed-method study, we analyzed the content of Dutch textbooks for reading comprehension instruction (i.e., the implemented curriculum) and teachers’ evaluation and use of these books (i.e., the enacted curriculum). A materials analysis of reading comprehension lessons (N = 80) in eight textbooks for grades 4 and 5 was complemented with semi-structured teacher interviews (N = 29) and lesson observations (N = 11), with a focus on the quality of reading strategy and text structure instruction in the curriculum. Main findings are (1) a lack of alignment between lesson goals, theory, and assignments, (2) a strong focus on practicing strategies, (3) limited declarative knowledge about strategies and text structure, (4) little opportunities for self-regulated strategy application. The teachers that were interviewed mention similar problems, but still hardly deviate from the textbook’s content and pedagogical guidelines. We make recommendations to improve the quality of the curriculum.

**Supplementary Information:**

The online version contains supplementary material available at 10.1007/s11145-021-10244-4.

Reading comprehension skills are crucial for children’s school success (Chall & Jacobs, 1983), and their future social lives and careers (Oakhill et al., 2015; Snow, 2002). Reading comprehension is however a multifaceted and complex skill that is affected by many underlying components. For instance, various studies have shown effects of prior knowledge (Smith et al., 2021), vocabulary breadth and depth (Beck et al, 1982), metacognitive skills (Soto et al., 2019), knowledge of reading strategies (Bimmel, 2001; Cain, 1999), and knowledge of text structures (Meyer & Ray, 2011) on reading comprehension.

The importance of reading comprehension skills calls for high-quality reading instruction, in which textbook quality is known to play a crucial role (Aaron et al., 2008; Wijekumar et al., 2021). In the Netherlands, teachers use specific reading comprehension textbooks, as reading comprehension is typically taught as a separate subject—apart from language teaching and technical reading (Garbe et al., 2016; van Gelderen & van Schooten, 2011). Where some countries (e.g., Austria, China, Portugal) specifically prescribe the core contents of the reading curriculum and sometimes even require schools to use textbooks that are explicitly approved by their government (Mullis et al., 2016; Zhang et al., 2021), the Dutch government only prescribes attainment targets in the so-called core objectives of the *Reference Framework for language and arithmetic* (Commissie-Meijerink, 2009). Therefore, educational publishers and schools can freely determine which reading strategies are taught and when (Garbe et al., 2016), and what pedagogical approach is used (Bruggink & Netten, 2017).

The quality of the current Dutch reading comprehension curriculum is under debate. Both national and international assessments show that a considerable group of Dutch students struggle with reading comprehension, both during primary education (e.g., PIRLS-2016: Gubbels et al., 2017; PPON-54: Kuhlemeier et al., 2014) and beyond (PISA-2015: Feskens et al., 2016; PISA-2018: Gubbels et al., 2019). The problem seems persistent and has not really changed over the past few years. In fact, a growing number of students are at risk of ending up as low literate (Pereira & Nicolaas, 2019). Also, Dutch students display the lowest levels of reading motivation in the world, and feel less involved in reading instruction than students in other OECD countries (Gubbels et al., 2017; Meelissen et al., 2012). Some institutions have therefore expressed their doubts about the quality of the curriculum, and recommend working with evidence-based pedagogies and materials to raise students’ motivation and performance in reading (Dutch Inspectorate of Education, 2019; Pereira & Nicolaas, 2019).

All in all, a gap arises between the intended curriculum (i.e., educational objectives) and the attained curriculum (i.e., students’ achievements) (Valverde et al., 2002). To some extent, this might be related to the quality of teaching materials. Textbooks form a crucial stepping stone between the intended and attained curriculum, and play a major role in the chain of effective instruction (Penuel et al., 2014). They affect teachers’ curriculum enactment and classroom practice (Ball & Cohen, 1996), also with respect to reading instruction (Aaron et al., 2008; Dewitz & Jones, 2013; Harwood, 2017).

According to the Component Model of Reading Comprehension (Aaron et al., 2008), ecological components, such as the quality of textbooks and teacher knowledge, affect ultimate reading achievement. Teaching materials constitute the *implemented* curriculum, which entails the learning content and instructional approach that teachers offer in their classrooms. Their quality and alignment with government objectives strongly determine effective education (Dockx et al., 2020; Penuel et al., 2014).

Given the importance of high quality textbook content for reading comprehension, a recent research initiative examined textbook content in various countries (Wijekumar et al., 2021). Key findings were limited attention for expository texts and text structure knowledge (e.g., Austria; Seifert, 2021; China: Zhang et al., 2021; Croatia; Peti-Stantić et al., 2021) and a strong focus on the recall of textual content in both instruction and student activities, often at the expense of promoting higher-order thinking strategies such as inferencing (e.g., Malta: Aguis & Zammit, 2021; Portugal: Cordeiro et al., 2021; US: Beerwinkle et al., 2021). The overall conclusion of this cross-country textbook analysis was that most textbooks do not sufficiently incorporate evidence-based practices (Wijekumar et al., 2021).

Although the aforementioned textbook studies provide valuable insights into textbook quality, teachers’ self-reported and actual use of these textbooks was not included, even though it is known that teachers moderate the effect of the teaching materials they use (Brown, 2009; Harwood, 2017), by selecting the materials they teach (Scheerens, 2017), but mostly by the level of pedagogical content knowledge (PCK) they can build upon in their enactment of these materials (Blömeke et al., 2014; Gudmundsdottir & Shulman, 1987). Even an excellent quality of teaching materials is therefore no guarantee that teachers are able to use them. This has also been shown in the area of reading comprehension instruction (Dewitz & Jones, 2013; Duke et al., 2011; Valencia et al., 2006). Therefore, textbook research needs to be complemented with an analysis of the *enacted curriculum* which refers to teachers’ actual use of these materials (Brown, 2009; Harwood, 2017), which is strongly affected by textbook quality (Valencia et al., 2006), and by teachers’ knowledge and beliefs (Ball & Cohen, 1996; Brown, 2009; Penuel et al., 2014).

Despite the debate on the quality of the Dutch reading curriculum (Dutch Inspectorate of Education, 2019; Pereira & Nicolaas, 2019), no recent studies have been conducted that closely examine both the implemented and enacted curriculum (Hoogeveen, 2018). There are indications that, in most schools, teachers still dutifully enact their textbooks, often without explicitly reflecting on learning goals and curricular structure (Scheltinga et al., 2013). This puts them at risk to copy flaws if textbooks lack quality (Aarnoutse, 1990). For example, some studies suggest that too much emphasis is put on question answering, at the expense of improving students’ reading process (Bonset & Hoogeveen, 2009; Rooijackers et al., 2020), and that both teachers and students often seem to consider reading comprehension as ‘answering questions about texts’, instead of as a crucial skill to gather knowledge through a written medium (Berends, 2012).

The aim of the current mixed-methods study is to carefully describe the features of teaching materials that are used for reading comprehension instruction in primary education in the Netherlands (the implemented curriculum), and examine how teachers experience and use these in their classrooms (the enacted curriculum).

Although the development of reading comprehension depends on a complex constellation of many different underlying skills, our current study puts emphasis on two aspects: text structure instruction and the teaching of reading strategies. Both text structure and strategy knowledge play a crucial role in theoretical models on reading comprehension such as the Landscape Model (van den Broek et al., 1999) and the Construction-Integration Model (Kintsch, 1988), and have been identified as powerful ingredients of comprehension instruction (e.g., Bogaerds-Hazenberg et al., 2021; Duke et al., 2011; Pyle et al., 2017). Text structure knowledge and reading strategies should therefore be well represented in curricular materials for reading comprehension (Wijekumar et al., 2021).

First, the Landscape Model theorizes that readers have to distribute their limited attentional resources over different concepts in a text to keep them activated, and to connect them to their prior knowledge. While building mental representations of the text out of these network activation patterns, readers need to monitor whether they are realizing their reading goals, or if they have a comprehension problem that needs to be resolved first. Metacognitive skills and sufficient knowledge about reading strategies are crucial to cope with comprehension problems (van den Broek et al., 1999). Therefore, reading strategies—mental tools that readers purposively use to monitor, repair, or support comprehension—often form a crucial part of reading curricula (Aarnoutse, 2017).

Teacher-directed reading strategy instruction can positively affect students’ strategy use and knowledge, and improve reading comprehension performance (e.g., Bråten & Anmarkrud, 2013; Brown et al., 1996; Houtveen and van de Grift, 2007; Palincsar and Brown, 1984). It therefore seems important to teach primary school students how to monitor and resolve comprehension problems by using a repertoire of evidence-based strategies, such as predicting information, self-questioning, summarizing, and inspecting text-structural features (Aarnoutse, 2017; Bimmel, 2001; Duke et al., 2011).

However, intervention studies do not always show substantial or even demonstrable effects of strategy instruction on standardized tests (e.g., Aarnoutse & Schellings, 2003; Andreassen & Bråten, 2011; Droop et al., 2016; Muijselaar et al., 2018). Besides methodological explanations (short interventions, test-related issues), it also seems that the quality of reading strategy instruction can break or make an intervention (Aarnoutse & Schellings, 2003; Andreassen & Bråten, 2011). For example, Okkinga et al. (2018) showed that the more elaborate explanations teachers provided on the nature, function, importance, and application of reading strategies, the better students performed on reading comprehension tests. Similarly, Droop et al. (2016) showed that investing in declarative knowledge about strategies without instruction in how and when to use these strategies does not directly pay off in terms of reading achievements for third and fourth graders. In other words, reading strategies should be taught in a way that helps students to become strategic readers who engage in a goal-directed, deliberate, and self-regulated use of these strategies (Afflerbach et al., 2008; Nash-Ditzel, 2014). One important suggestion is therefore that high-quality reading strategy instruction should enable students to develop three types of knowledge (Cross & Paris, 1988; Kostons et al., 2014; Paris et al., 1983):

- Declarative knowledge: factual knowledge about strategies, text structures, and genres, needed for adequate goal setting and strategy use;
- Procedural knowledge: knowing and reflecting on how to apply reading strategies;
- Conditional knowledge: when-and-why knowledge needed to match reading behavior to the task or text at hand (closely related to metacognitive knowledge and skills).

The more knowledge students obtain about how different reading strategies operate in various situations (e.g., reading purposes, text structures), the better they will adapt their reading behavior to a given situation, both inside and outside the direct context of comprehension lessons (Afflerbach et al., 2008). For example, a reading strategy like skimming is useful to read a list of online search results, but not for reading poetry. Without sufficient conditional knowledge, students are unable to match their use of reading strategies to the text at hand, lack insight in the value of reading strategies, and cannot become self-regulated strategic readers (Nash-Ditzel, 2014; Simpson & Nist, 2000; Taboada & Guthrie, 2004).

Second, teaching materials should include instruction about text structures, or the ‘organization of ideas, the relationship among the ideas, and the vocabulary used to convey meaning to the reader’ (Pyle et al., 2017, p. 1). According to the Construction-Integration Model of text comprehension (Kintsch, 1988), readers parse text input into concepts and relationships which they need to organize in associative networks. They need to make connections between ideas within the text, and with prior knowledge, until they arrive at a coherent mental representation of the text: the situation model (Kintsch, 1988). Text structure can facilitate the construction of a situation model, as good readers do not simply use a default list-strategy to organize information, but instead pick up text-structural cues and use these to organize main ideas accordingly (Meyer et al., 1980).

It has been shown that knowledge about text structures helps students to locate, recall and interconnect certain pieces of information better (Meyer & Ray, 2011). Explicit text structure instruction relates to better comprehension, recall, and summarization skills (Bogaerds-Hazenberg et al., 2021; Hebert et al., 2016; Pyle et al., 2017). Therefore, a high-quality reading curriculum should also include explicit text structure instruction that enables students to acquire declarative knowledge about different text structures (e.g., on the rhetorical goal of each structure and on its defining signaling words: *however, both*), and procedural knowledge (e.g., how to recognize different text structures)*.* Knowledge about text structure also matters for conditional knowledge, because an increased awareness of different informational patterns of text can facilitate a purposeful and context-sensitive application of reading strategies. For example, the main idea of compare-contrast texts is phrased differently than that of a cause-effect text (Hoch & McNally, 2020; Oakhill et al., 2015).

In sum, teaching materials should offer a broad spectrum of declarative, procedural and conditional knowledge about, and room for practice with reading strategies, and provide explicit text structure instruction. Furthermore, they should provide students with ample opportunities to deliberately plan, monitor, and evaluate their strategy use in increasingly complex texts, until these overt strategies eventually transform into covert skills as readers use them more or less automatically (Afflerbach et al., 2008). In this study we therefore focus on the question to what extent this declarative, procedural, and conditional knowledge is realized in Dutch teaching materials for reading comprehension (the implemented curriculum), and how teachers enact and evaluate this (the enacted curriculum).

## Materials and methods

In order to describe the Dutch reading comprehension curriculum (i.e., textbook quality, and teachers’ perspectives and enactment of textbooks), we triangulated data from three different methods, thereby increasing the validity of our study (Table 1; Brown, 2009; Kuorikoski & Marchionni, 2016; Miles et al., 2013): a materials analysis, teacher interviews, and lesson observations that all focused on similar topics, such as curricular structure and lesson goals, characteristics of instruction, characteristics of student activities, and transfer and metacognitive skills.^1^

**Table 1:** Overview of sources for mixed-methods analysis per textbook

Textbook	Materials analysis			Teacher interviews	Lesson observations
	Info brochure	Teacher manual	Textbooks, work sheets		
*Bliksem (BL)* (Bazalt, HCO, Expertisecentrum NL, 2013)	√	√	√	4	0
*Grip op lezen (GL)* (Malmberg, 2012)	√	√	√	4	2
*Kidsweek in de klas (KW)* (Young & Connected, 2009)		√	√	3	0
*Leeslink (LL)* (Malmberg, V-2020)	√	√	√	3	1
*Lezen in beeld (LB)* (Zwijsen, 2009)		√	√	4	0
*Lezen* = *weten (LW)* (Bazalt, 2011)	√	√	√	3	0
*Nieuwsbegrip XL (NB)* (CED-groep, V-2017)	√	√	√	4	6
*Tekst verwerken (TV)* (Noordhoff, 2006)	√	√	√	4	2

### Materials analysis

We made an inventory of the content of eight textbooks specifically designed for reading comprehension instruction (Table 1). Together, these books cover the vast majority of the Dutch curriculum and exemplify the diversity of the curricular landscape, as we included textbooks with very large market shares (e.g., *Nieuwsbegrip)* as well as two niche methods (*Bliksem, Lezen is weten).* In all textbooks, students learn and practice comprehension strategies related to different phases of reading (before, during, after reading).

Per textbook, ten instruction lessons for grades 4 and 5 were randomly selected (N = 80; 12–18% of the year curriculum). The textbook analysis was complemented with an analysis of teacher manuals that typically contained the full blueprint for each lesson (e.g., modeling scripts, instructional approach), informational brochures highlighting the intentions of the publishers, and student work sheets. All materials were analyzed with a coding scheme that focused on content features of the lesson goals, theory and assignments. This was a recursive process in which extra categories were added to the initial coding scheme if necessary (Miles et al., 2013). Per lesson, we analyzed whether lesson goals, theory and instruction, and student activities related to declarative, procedural, and/or conditional knowledge.

- Declarative knowledge: we coded the presence of factual knowledge about strategies (e.g., a list of reading strategies), explanation about the features of global text structures (e.g., cause-effect) and genres (e.g., a recipe), or about local text structure (e.g., signaling words). For lesson goals, this applied if students were supposed to *know* – not *do* – something (e.g., ‘You *know* the strategies that can be applied before reading’). We also counted how many assignments related to global or local text structure, or genre.
- Procedural knowledge: we coded the presence of information on how to apply strategies or knowledge on text structures (e.g., *how to* scan a text). For lesson goals, this applied if students were supposed to *know how* to do something (e.g., ‘You *know how* to find a main idea’). We also coded the type of strategy (before, during, or after reading), counted how many student assignments were provided, and whether assignments were aligned with lesson goals (e.g., lesson goal on summarization, assignment asking students to select main ideas).
- Conditional knowledge: we coded whether information was provided on how to apply reading strategies in other contexts, whether students could plan and evaluate their strategy use, and if there was attention for transfer (e.g., applying strategies in content-area texts). Lesson goals were classified as conditional if students were supposed to learn *when* or *why* strategies work (e.g., ‘You know *when* you use a Venn diagram to summarize a text’). Per lesson, we counted how many questions related to conscious strategy planning and evaluation, transfer, and explicit conditional knowledge (e.g., ‘What type of text would you summarize with a timeline?’).

Most knowledge aspects were coded as dichotomous variables (present/not present in lesson); only assignments and questions were counted as we wanted to examine the amount of practice. The reliability of the materials analysis was increased by discussing difficult cases during meetings with all authors (Lincoln & Guba, 1985). A subsample of 16 lessons (2 per textbook) were also coded by a second coder, resulting in sufficient interrater agreement percentages (Miles et al., 2013). For coding of variables related to declarative, procedural, and conditional knowledge, the mean interrater agreement percentages were 87% (min = 69%, max = 100%), 79% (min = 69%, max = 100%), and 91% (min = 75%, max = 100%) respectively. Most aspects resulted in high agreement percentages, and for the other aspects, minor differences were resolved in discussion between the two raters.

### Teacher interviews

In order to gain insight into teachers’ enactment and appreciation of the textbooks, 29 semi-structured interviews were conducted with mainly fourth and fifth grade teachers (86% female, M_experience_ = 16.4 years, M_age_ = 39.7 years), who had been working for at least two years with their textbook (M_methodinuse_ = 4.3 years). Teachers were recruited from 29 schools throughout the Netherlands. The distribution in denominations (9 Catholic, 14 Public, 6 Protestant) and demography (eight different provinces; 50% of schools in large towns) was representative for the Netherlands.

Interview questions covered the same topics as the materials analysis and lesson observations: curricular structure and goals, theory and instruction, student activities, and transfer, but with a focus on teachers’ actual teaching practice and satisfaction with the textbooks. For each topic, teachers were asked about their actual teaching practice (e.g. for transfer, ‘Do you strive to integrate reading comprehension lessons with other subjects? How exactly?’), and to evaluate their teaching materials with respect to this topic (‘To what extent do the teaching materials support you in doing so?’). All interviews were audio-taped and transcribed with permission of the participating teachers. Verbal data were then inspected for emerging patterns and compared to findings from the materials analysis and observations (Lincoln & Guba, 1985).

### Lesson observations

With permission of school leaders and teachers, reading comprehension lessons were observed by one of three observers. We strived for three lesson observations per teaching method, and six for *Nieuwsbegrip,* as this teaching method is strongly in vogue in Dutch schools, but due to COVID-19 restrictions, we completed only eleven lesson observations (Table 1). We recruited a different group of teachers for the lesson observations to increase our sample size; only one of the observed teachers also participated in an interview. The participating teachers taught in grades 4 and 5 or in mixed grades (4–6) and were all qualified to teach (83% female, M_experience_ = 9.3 years). Their schools were located in two central provinces (36% schools in villages or small towns) and most schools were religiously affiliated (3 Catholic, 2 Protestant, 2 public schools).

Observers coded whether certain predetermined features of the lesson content occurred, for example with respect to lesson goals: ‘Teacher mentions lesson goal’ or ‘Teacher discusses when/why attainment of lesson goal is important’. These features covered more or less the same topics as the materials analysis and interviews, but provided additional insight on specific pedagogical aspects of lesson execution (e.g., ‘Teacher provides process/product feedback’). Observers also noted how much time was devoted to different lesson phases (e.g., teacher-led instruction, individual question answering). Due to strict ethics regulations, lessons could not be videotaped, but the validity of the observation scheme was increased by formulating concrete events that were evaluated on occurrence and duration, and by leaving room for a general impression and for remarks, so that difficult decisions could be discussed afterwards (Miles et al., 2013).

## Results

The following sections provide an overview of the implemented curriculum in textbooks, followed by a discussion of the enacted curriculum, for each type of knowledge separately. The two-letter abbreviations behind textbook and teacher quotes in this section show to which textbooks these quotes refer to (see Table 1).

### Declarative knowledge

#### Implemented curriculum

All textbooks pay relatively little attention to declarative knowledge (Table 2). In 19% of the analyzed lessons, a declarative knowledge goal is formulated (‘After this lesson you know that…’). In lesson goals and theory, the main focus is typically not on declarative strategy knowledge, but on strategy application. Six textbooks provide review or integration lessons in which all reading strategies are repeated. However, these lessons consist of extensive practice, without explicit strategy instruction; declarative knowledge is often restricted to mentioning that students can consult the Appendix for an overview of all strategies while working on questions. *Lezen in Beeld* pays slightly more attention to declarative knowledge than the other textbooks, and also asks students to memorize the different reading strategies and the order in which they should be applied.

**Table 2:** Materials analysis: overview of declarative knowledge results

Unit of analysis		Yes	%	Example
Goals	Among general objectives	1		Students learn the purpose, structure, and lay-out of many types of texts
	Declarative lesson goals	5	19	You know the purposes of advertising
Theory	Explicit review of all strategies (≥ 4)	5	13	You learn to apply all six key strategies
	Text structure	3	10	There are fixed structures, like question–answer, cause-effect, then-now
	Signaling words	6	13	‘So’ is a signaling word for conclusion
	Basic division intro-body-conclusion	4	5	A text has an introduction, a body and a conclusion. Paragraphs have white lines
Exercises	Genre, local or global text structure	8	44	What are the features of a report? What is the structure of paragraph 1?

Yes = present in number of textbooks; % = percentage of all analyzed lessons

Textbooks differ from each other most notably in their text selection. In most lessons, *Kidsweek* (KW), *Grip op Lezen* (GL), and *Lezen in Beeld* (LB) offer a relatively rich variety of texts (i.e., varying genres and lengths). *Nieuwsbegrip* (NB) and *Leeslink* (LL) put great emphasis on news articles and provide weekly updated texts. The niche method *Bliksem* (BL) focuses primarily on children’s literature, and *Lezen is Weten* (LW) prompts teachers to make use of expository content-area texts. However, the attention devoted to declarative knowledge about genres and text structures is quite limited across textbooks. One textbook explicitly lists knowledge on various genres and text structures among its global learning objectives.

Concerning text structures, 13% of all analyzed lessons pay some attention to signaling words, 5% to the introduction-body-conclusion division, and 10% of the lessons mention text structures (e.g., chronology, problem–solution, story grammar), although this only happens in three textbooks. Most declarative knowledge on text structures is restricted to definitions, as in (1) and (2).

1. Cause-effect signaling words tell what causes something (e.g., *therefore, as a result*). (GL-4)

2. A text has an introduction, a body, and a conclusion. (TV-4)

Some textbooks provide more elaborate but still relatively abstract explanations. For example, different types of signaling words are mentioned without explaining in what structures or genres students can expect these signaling words, and how they can facilitate comprehension. Only *Leeslink* explains to students not only which signaling words figure in chronological texts, but also *why* this is useful to know (3):

3. A text does not always discuss events in their original order. If you make a list of all events ordered in time, you will understand the text better. Signaling words can help you to do this. (LL-5)

While textbooks offer limited information on text structures, a relatively large proportion of student assignments across textbooks is devoted to text structure (44%). These questions prompt students to recognize genres, structures, and signaling words (4), to complete graphic organizers, or to locate the introduction, body, and conclusion of texts (5).

4. He hasn’t been there for years, ____ he notices many changes in his country.

Fill in: A. Because B. Therefore C. So that D. Hereby (GL-4)

5. What parts of the text are introduction, body, and conclusion?

The introduction is in paragraph ____.

The body is in paragraph ____ to ____.

The conclusion is in paragraph ____. (TV-4)

#### Enacted curriculum

Declarative knowledge goals are lacking in all of the observed lessons. Instead, most teachers closely follow the guidelines in the textbook, except for the fact that they often add activities related to the topic of the specific text (e.g., video clip on ocean pollution). Four teachers (36%) briefly discuss aspects related to genre or local text structure while providing feedback on assignments (e.g., discussing a few signaling words of time), but they never mention global text structures (e.g., cause-effect).

A similar picture arises from the interview data. The teaching of reading strategies is recognized as important content, while none of the teachers spontaneously mention text structure or genre knowledge as important content. Three teachers even intentionally skip declarative knowledge on genres and signaling words, because they do not feel that it matters for text comprehension, or because they experience difficulties explaining topics related to text structure (6).

6. I find it very hard to explain signaling words to my students; in fact, I find it even too hard. (…) To be honest, I don’t understand why my students should know about signaling words. I don’t think it is important. They simply need to understand the text! (TV-teacher)

By contrast, one third of the teachers (34%) mention that their textbook does not provide sufficient opportunities to teach about topics related to global and local text structure (e.g., summarization, signaling words, referential coherence), and that they experience difficulties teaching it without guidance of their textbook (7). This was mentioned by teachers from all textbooks. Interestingly, the LW-teachers only reported issues with local text structure topics (e.g., referential coherence), but were very positive about topics related to global text structure (e.g., summarization and schematizing). Some teachers (14%) remediate the lack of attention for text structure by giving their students extra texts with questions focused on local text structure (i.e., signaling words and referential coherence); to a much lesser extent by providing additional instruction themselves.

7. There is little on summarization. (…) My students also struggle with signaling words and referential pronouns. There are too few materials about it. (LB-teacher)

Still, most interviewed teachers (76%) do not adapt the curricular content, but try to enact all lessons with dutiful fidelity. In fact, for most teachers, the central consideration in their choice or appreciation of a textbook is not the curricular quality (14%), but rather whether the texts will capture students’ interest and keep them motivated to read (34%), and whether the textbook has an attractive lay-out (21%). Teachers (38%) across all textbooks explicitly mention that they are unsure about the curricular structure, except for the LW-teachers who have to develop the curriculum more or less themselves. Some teachers simply trust their textbook (8), others feel less positive about this, even though they don’t make changes (9).

8. I don’t see a clear structure [in the curriculum], but I believe that what the textbook offers is what children need to know. (TV-teacher)

9. We want to let go of it [the strict curriculum], and be more adaptive to children’s needs. But we cannot do that as long as we don’t know what is needed for good reading comprehension. (LL-teacher)

### Procedural knowledge

#### Implemented curriculum

In all textbooks, emphasis is put on procedural knowledge, especially on practicing reading strategies (Table 3). All brochures mention a fluent use of strategies as their main objective. In all textbooks, a fixed set of reading strategies dictates the curricular structure and lesson goals.

**Table 3:** Materials analysis: overview of procedural knowledge results

Unit of analysis		Yes	M/%	Example
Goals	Among general objectives	8		Students learn how to use each of the seven evidence-based reading strategies
	Procedural lesson goals	8	91	You create a flow chart; You know that you should scan a text before reading
Theory	Strategies before reading	8	31	First, figure out why you want to read the text, e.g., to learn about a topic
	Strategies during reading	7	32	If you don’t know the meaning of a word, read the text again or look at the pictures. Or you can use a dictionary
	Strategies after reading	8	37	In an arrow scheme you can write down the main idea and all the subtopics
Exercises	Mean number of exercises	8	11.3	
	Related to theory/goal	8	59	

Yes = present in number of textbooks; % = percentage of all analyzed lessons; M = mean number in all analyzed lessons

Procedural lesson goals appear in 91% of the analyzed lessons (‘After this lesson you are able to…’), most often as the only goal of that lesson (68%). They are evenly distributed over the phases of the reading process: before (31%), during (32%), or after (37%) reading. In some textbooks, the wording of lesson goals focuses more on the central activity than on the procedural knowledge to be acquired (10), or foregrounds text content (11).

10. In this lesson you create a flow chart of the text. (TV-5)

11. You will learn about an owl who learnt to fly with donor feathers, and how making use of prior knowledge can help you. (KW-4)

Even though most textbooks lack content-related lesson goals (11), they still put an emphasis on text content. For example, 16 lessons (20%) prescribe a lesson start with video clips and/or extensive discussions about the text topic, without linking it to the lesson goal (12). As a result, no or much less instructional time is left for actual strategy instruction.

12. Does anyone play soccer? What is so fun about playing soccer? What is your favorite club? What is your parents’ favorite club? Why? (…) (TV-5)

The emphasis on procedural knowledge is also reflected in theory and exercises: in 80% of the analyzed lessons, the theory and instruction concern procedural knowledge and describes how certain reading strategies should be applied. In comparison to the other textbooks, *Kidsweek* provides quite elaborate procedural knowledge about the different reading strategies, often with step-by-step instructions. Except for the two niche methods*,* textbooks expect students to answer a large number of questions about the text each lesson (*M*_overall_ = 11.3, *M*_niche methods excluded_ = 15.1, max = 31). On average, 59% of the tasks or questions per lesson are directly related to the lesson goals and theory.

#### Enacted curriculum

Most interviewed teachers (76%) believe that using reading strategies correctly is the main goal of comprehension instruction, and that this is also the primary objective of their textbook and the focus of their lessons. Several teachers (28%) also consider vocabulary growth and acquiring world knowledge as important objectives: their students should have learned the text content upon lesson completion (13). To this end, about half of the teachers (48%) say that they often (re)read the text together, and/or extensively discuss students’ personal experiences and prior knowledge (38%), or even answer all questions together (17%).

13. Of course, there are those reading strategies, and we do practice them, but it should all be about the text. My lesson goal is always something like: ‘I know why a boat sank in the Mediterranean Sea.’ Such lesson goals are more fun. (KW-teacher)

Three teachers mention that their students often remember the text topic of the previous lesson, but not the reading-related learning goals. One teacher attributes this to poor alignment of lesson goals, theory, and exercises (14).

14. You have to introduce the lesson goal first, then you discuss the text, and start answering questions. But only halfway the lesson, you get to the questions that relate to the goal (…). They give them almost no opportunity for practice. (TV-teacher)

Lesson observations show that six teachers (55%) do not mention the lesson goal, even though their textbooks provided explicit lesson goals, and only three teachers (27%) actually provide explicit instruction related to the lesson goal. Instead, most teachers (82%; nine teachers) mainly focus on activating prior knowledge on the text topic and building vocabulary. To this end, three teachers show video clips (27%), five teachers create mind maps (45%), and most teachers also extensively discuss students’ experiences or knowledge on the topic (64%; seven teachers). Three teachers (27%) explicitly add their own content-related lesson goal (e.g., ‘By the end of this lesson, you can mention the advantages of 3D printers’). Also, strategy practice is an important ingredient of reading comprehension lessons: in all of the observed lessons, most lesson time (42%) is focused on individual question answering.

Most interviewed teachers (69%) display a positive attitude towards fostering procedural knowledge through extensive practice, and seem satisfied with their textbook’s focus on comprehension questions. Some teachers (17%) say that they answer almost all questions together with their students; during the observed lessons, even more teachers (36%; four teachers) appear to do this. The value teachers attach to question answering might be one of the reasons why teachers closely follow their textbooks (15). Four teachers even adapt their curriculum by providing additional texts with traditional question answering (16).

15. I never use other texts, because then I would have to make up the questions myself (…). The questions help my students to think about what they are doing. (LB-teacher)

16. We were so busy practicing those reading strategies, that my students no longer really read texts. So I select texts from another old-fashioned book with simple question-and-answer tasks, and they learn so much more now! (LL-teacher)

At the same time, various teachers (34%) complain that their students rush through the extensive number of exercises, or that students get bored, especially while working on their own (17). Some of the interviewed teachers (27%) also regret that they often lack time to discuss answers and provide feedback.

17. They find texts and questions too long. Children are too lazy to finish reading the whole text. To me it seems that they are lazy and bored. So in fact, they stop focusing on comprehension during comprehension lessons! (LB-teacher)

Nevertheless, question answering is the main focus in most of the observed lessons (73%), almost as a goal in itself, as shown in a quote by one of the teachers (18). Students’ answers to comprehension questions are often only briefly discussed with little attention for the underlying process of text comprehension (64%). Only two teachers (18%) exploit this as an opportunity to provide additional instruction.

18. Even if you have to read the text and the question ten times, you just have to do it. You read the text over and over again, until you know the right answer. (NB-teacher)

Irrespective of the textbook they use, interviewed teachers (34%) feel obliged to have their students practice with question types that typically appear in Cito tests: the national assessments of reading comprehension. In one of the observed lessons, all instruction and practice focuses on answering Cito multiple-choice questions. For many interviewed teachers, Cito tests have become the gold standard; teachers criticize the quality of their textbook for not being sufficiently aligned with the types of questions and texts in Cito tests.

### Conditional knowledge

#### Implemented curriculum

It is important that students learn to consciously plan, regulate and evaluate their use of reading strategies. Conditional knowledge is needed for this skill to develop, but the teaching materials pay little attention to this type of knowledge and hardly explain under what circumstances a certain strategy is most powerful (Table 4). Only 5% of the analyzed lessons contain an explicit conditional knowledge goal (‘After this lesson you know when/why…’). In 39% of the lessons, textbooks provide some theory about why or when a reading strategy is useful, but this explanation mainly concerns a general statement about the usefulness of a strategy, such as ‘This will help you to understand the text.’

**Table 4:** Materials analysis: overview of conditional knowledge results

Unit of analysis		Yes	M/%	Example
Goals	Conditional lesson goals	2	5	You understand why you should make use of prior knowledge while reading
Theory	Conditional knowledge	7	39	This strategy will help you understand a text better
Exercises	Mean number of exercises on planning and evaluating strategy use	6	0.69	How well did you understand the text? What would you do differently?
	Mean number of transfer exercises	6	0.68	Write a short text about animals. Do not use referential pronouns. Now read your story. Do you like how it is written?
	Suggestions for transfer in teacher manual	6	53	When you read other texts, ask students if these are about facts or opinions
	Transfer tasks aligned with goals	6	35	Text about art: “Close your eyes. How do you feel? Express it in a painting”

Yes = present in number of textbooks; % = percentage of all analyzed lessons; M = mean number in all analyzed lessons

Students are almost never at liberty to choose and motivate which reading strategy they want to apply, because exercises dictate students what to do—as in (19)—or because most lessons focus on one strategy only. Even in so-called integration lessons in which students are supposed to review multiple reading strategies, they are almost never asked to plan and evaluate their own strategy use.

19. Read the title of the text. Look at the pictures. What do you think is the topic of the text? About ________________________. (LL-4).

A positive exception to this pattern is the niche method *Bliksem.* This teaching method focuses more on conditional knowledge than the other textbooks, by having children apply multiple reading strategies on self-selected text passages, and evaluate their usefulness. Only six percent of all analyzed questions tap into conditional knowledge (M = 0.67 per lesson), by asking students to evaluate if a certain strategy matched their reading goals (20), or—to a lesser extent—plan their strategy use (21). As most of these questions are part of independent practice, students might not receive feedback on their response.

20. How well did you understand the text? What would you do differently next time? A. Reread parts B. Read slower C. Take notes D. Other: …. (NB-5)

21. When would you make a summary: while reading a book for pleasure, or while studying for a history test? Why? (BL-5)

Although most textbook brochures mention that transfer is of paramount importance, lesson plans offer little opportunities for students to apply reading strategies in other contexts (e.g., content-area subjects). Attention to transfer often consists of suggestions for teachers to model a specific strategy during other lessons (53%). Some textbooks contain tasks other than comprehension questions (e.g., aimed at writing, speaking, or reading content-area texts) that might promote transfer. Such transfer tasks are limited (*M* = 0.68), and are often presented as optional assignments, especially for high-ability students. These tasks focus on writing and speaking (43%), the reading of content-area texts (35%), or involve creative exercises, such as making a drawing related to the text topic (22%). A closer inspection shows that 65% of these tasks are not related to the learning goal, but to the text topic and cannot be classified as true transfer promoting tasks. For instance, a lesson with a text on shipping also contains a task in which students write a captain log book and make a drawing, which was unrelated to the lesson goal.

#### Enacted curriculum

More than a quarter of the interviewed teachers (28%) express doubts related to the development of conditional knowledge. For instance, they wonder if textbooks provide students with the knowledge needed to plan and evaluate their reading process (22 and 23). At the same time, some teachers (21%) are negative about lessons in which students are asked to apply multiple strategies more independently. They often skip these lessons, as they are afraid that their students are ill-prepared and would get overwhelmed. By contrast, the teachers who use the niche method *Bliksem* are enthusiastic about the way in which their textbook promotes metacognitive skills.

22. In most textbooks, the focus is too much on writing down answers to a series of questions, and too little on critical thinking. (BL-teacher)

23. There is little room for students’ own initiative. For example, supposedly difficult vocabulary is already underlined and explained. They do not let students come with their own questions and solutions. (KW-teacher)

This lack of conditional knowledge is also apparent in the observed lessons: only one teacher explains when and why it is useful to summarize a text. None of the other teachers explicitly discuss when or why the lesson goal or theory of that lesson could be useful. For instance, in one lesson, the teacher provides excellent instruction on how to use a dictionary, without ever mentioning in what situations a dictionary search is useful, so that students might end up with the wrong idea that you have to look up every single difficult word you encounter in a text, while less effortful strategies could be used as well. Most teachers follow their textbook and the clear-cut student exercises, but two teachers (18%) provide their students with more freedom to apply multiple strategies while reading, by asking them to circle and search the meaning of unknown words, and to highlight key information.

Almost all of the interviewed teachers find it important that their students apply their knowledge of reading comprehension in contexts outside reading class. Therefore, teachers try to review some reading strategies during content-area (58%) and language lessons (31%), and some during arithmetic (10%) or writing instruction (7%). This attention to transfer is often spontaneous and typically restricted to simply mentioning an appropriate reading strategy or pointing at a list of strategies on a poster, instead of modeling a specific strategy in a new context. Teachers (38%) quite often mention that they skip the transfer exercises provided by their textbook, because they consider them as optional, or feel not confident to teach them (24).

24. We find it difficult to apply strategies during content-area lessons, because you should select texts yourselves and look for a suitable strategy. (…) We tried to do it for geography, but we don’t feel confident to teach without a manual. (BL-teacher)

## Discussion

The ecological component of reading comprehension is a complex construct, suggesting that students’ achievements in reading are at least partially affected by teacher knowledge and the quality of textbooks (Aaron et al., 2008). Therefore, our study focused on what textbooks offer and what teachers teach during reading comprehension. In order to gain insight into the quality of the Dutch reading comprehension curriculum, we used a mixed-methods analysis to examine both the layer of the implemented and enacted curriculum, as teachers moderate the effect of the teaching materials they use (Brown, 2009; Harwood, 2017). Our study reveals various knowledge aspects of reading strategy and text structure instruction that could be improved in the different layers of the current Dutch curriculum. Despite individual differences between teachers, we found robust patterns in the implemented and enacted curriculum, that were confirmed by methodological triangulation.

The main findings from our study are as follows. First, we found that the implemented and enacted curriculum are quite similar: most of the interviewed and observed teachers follow the curricular content linearly, despite complaints about the clarity or usefulness of the curriculum, thereby copying the poor alignment between lesson goals, theory and exercises in their classroom. Only a few teachers explicitly wished they had more knowledge to flexibly adapt their instruction to students’ needs and be less dependent on their textbooks.

This has been reported in previous studies as well: teachers with limited pedagogical content knowledge often follow their materials with ‘dutiful fidelity’ (Beerwinkle et al., 2018; Dewitz & Jones, 2013; Valencia et al., 2006; Wijekumar et al., 2019) and are at risk to copy flaws (Aarnoutse, 1990). By contrast, self-confident and knowledgeable teachers critically examine, flexibly adapt, and actively evaluate the right sequence of instruction, lesson plans, and activities (Jitendra et al., 2001; Penuel et al., 2014; Piasta et al., 2009).

Second, although reading researchers have advocated a balanced focus on declarative, procedural, and conditional knowledge to teach reading strategies, our study shows how an imbalance between these types of knowledge in the curriculum might lead to various problems. In the materials that we studied, there is a strong emphasis on the extensive practice of so-called ‘evidence-based’ reading strategies (e.g., questioning, making predictions) (Aarnoutse, 2017; Bimmel, 2001; Duke et al., 2011). Procedural lesson goals and texts-with-questions predominate the textbooks, so that much lesson time is devoted to answering comprehension questions. Due to this strong emphasis on procedural knowledge in the Dutch curriculum, it seems as if strategies have become a goal in itself rather than a means to an end.

To some extent, practicing reading strategies is good; it can turn these strategies eventually into effortless skills that can flexibly be used to support comprehension (Afflerbach et al., 2008), and, for teachers, comprehension questions are easy to implement and facilitate student monitoring. However, experimental research suggests that the text-question–answer model has limited effects on reading comprehension skills (Rooijackers et al., 2019). Moreover, in the materials that we studied, many questions turned out to be unrelated to lesson goals and theory. As a result, students might ignore the purpose of these questions and lack the necessary knowledge for task execution.

Some interviewed teachers noticed negative effects of this lack of alignment: students rush through the numerous questions and often remember the text topic, but not the intended lesson goal. Unfortunately, the observed teachers seemed to copy the lack of alignment in their classrooms: they often did not explicate the learning goals—even though their textbooks provided these—and strongly focused on text content and right answers. This makes it questionable if students actually internalize the intended reading strategies.

Although some of the interviewed teachers criticize the text-question–answer model, it still dominates reading comprehension lessons. This problem might be amplified by a negative backwash effect of the testing culture in the Netherlands where much value is attached to standardized reading tests (Bartels et al., 2002). Instead of such tests being designed at the service of learning and teaching, teaching has become at the service of testing (Hamp-Lyons, 1997), thereby undermining the instructional time devoted to higher-order thinking skills (Cheng & Curtis, 2004).

In other words, the strong procedural focus on reading strategies in Dutch textbooks—and as a result also in actual classroom practice—has made these strategies an end in themselves, rather than a means to an end. However, for effective strategy instruction, it is fundamental to pay attention to the underlying process of goal-directed decision making on the right reading approach in a given situation (Afflerbach et al., 2008; Paris et al., 1983). Instead of the dominant text-question–answer model, students might benefit from well-aligned, functional reading tasks as a context for strategy practice, and from high-quality instruction on the nature, function, importance, and application of reading strategies (Okkinga et al., 2018).

This is directly related to the third main finding: our analysis of Dutch textbooks and classroom practice revealed very limited attention to conditional knowledge development. Conditional knowledge is developed when students know when, where, and why strategies should be applied, and when they can independently choose and evaluate what strategy they want to apply in a given situation (Afflerbach et al., 2008; Simpson & Nist, 2000). The textbooks in our sample often lacked explanation on *when* or *where* a given strategy should be applied, and only one of the observed teachers explained when the strategy she was demonstrating could be useful. The textbooks and teachers in our sample also provided very little opportunities for students to plan and evaluate their strategy use. Textbooks often focused on one or two strategies per lesson, and questions quite specifically dictated what strategy should be applied next. Some of the interviewed teachers intentionally skipped lessons that would require a more independent use of strategies, because they consider such integration lessons as too difficult.

This problem might be even more amplified due to the fact that, in the Netherlands, the different skills underlying reading comprehension are taught in isolation, and reading comprehension is taught as a separate subject. This is often perceived as demotivating (Berends, 2012), and makes the transfer of reading strategies to other subjects more challenging (van Gelderen & van Schooten, 2011). Similar problems have been reported in other countries: reading skills are often taught in isolation (Wijekumar et al, 2019) with very limited attention to conditional knowledge (Snow, 2002), even though developing readers should learn to be metacognitive, by deciding which strategies work best in which context (Afflerbach et al., 2008; Simpson & Nist, 2000; Soto et al., 2019) and by learning to coordinate multiple strategies (Reutzel et al., 2005). Teaching skills in isolation without authentic opportunities for transfer might therefore be an unfortunate approach.

The declarative knowledge component is also quite shallow in the Dutch textbooks we studied, especially when it comes to text structures, even though explicit text structure instruction relates to better comprehension, recall, and summarization skills in primary school (Bogaerds-Hazenberg et al., 2021; Hebert et al., 2016; Pyle et al., 2017). A similar lack of attention to text structures in textbooks has been reported in other countries (e.g., Austria: Seifert, 2021; China: Zhang et al., 2021; Malta: Aguis & Zammit, 2021; US: Beerwinkle et al., 2018, 2021; Wijekumar et al., 2019, 2021). A striking similarity with these studies is the fact that if text structure is discussed, it is often not made explicit to students why and how knowledge of text structure is needed to gain a deeper understanding of the text, which underscores again the underrepresentation of conditional knowledge.

The Dutch teachers that we interviewed seemed relatively unaware of this gap and did not add their own materials on text structures. In fact, most teachers did not feel that text structure is an important topic, although some teachers complained that their students struggle with questions related to text structure. The teachers in our sample try to fix these problems by providing their students with additional practice—not explanation. This might indicate that teachers themselves also lack the knowledge to provide high quality instruction on text structure, as has been reported in many studies outside the Netherlands as well (e.g., Beerwinkle et al., 2018; Dockx et al., 2020; Seifert, 2021; Wijekumar et al., 2019, 2021).

All of these factors contribute to the Peter Effect: teachers cannot give what they do not have (Applegate & Applegate, 2004; Beerwinkle et al., 2018). Even though we did not assess teachers’ general knowledge and beliefs about reading comprehension, it seems as if teachers hardly compensate for suboptimal textbook content in their classroom enactment. This only underscores why it is crucial to invest in both the quality of textbooks (Dewitz & Jones, 2013; Harwood, 2017; Pereira & Nicolaas, 2019; Valencia et al., 2006), and in teachers’ pedagogical content knowledge (Gudmundsdottir & Shulman, 1987; Piasta et al., 2009; Wijekumar et al., 2019). For example, by training teachers in strategy instruction (Okkinga et al., 2018), and by including more knowledge about text structures in the Dutch teacher training curriculum (e.g., Kooiker-den Boer et al., 2019), so that teachers might gradually become less dependent on the quality of their textbooks, and will flexibly adapt their teaching to their students’ needs.

Of course, our study has its limitations. First, although we strived for varied data sources equally representing all eight different textbooks, our observational data overrepresent *Nieuwsbegrip* due to COVID-19 restrictions, which might limit the generalizability of our observational findings. A second limitation is the fact that we interviewed a sample of only 29 teachers. We already increased the external validity of our study by recruiting different groups of teachers for the observations than for the interviews, but the downside of this approach is that it becomes challenging to make inferences about the exact sources of (dis)agreements between the implemented and enacted curriculum. Importantly, the observations often still confirmed findings from the interviews and materials, in which all textbooks were equally represented. Moreover, these findings seem not to be restricted to the Dutch context, but also resonate findings from other international studies on textbook quality (Beerwinkle et al., 2018; Seifert, 2021; Wijekumar et al., 2021; Zhang et al., 2021).

Given the limitations, it would be worthwhile to examine in future research whether our findings can be replicated and elaborated. For example, by adding student-level data to examine the impact of textbook and teachers on the ultimate attained curriculum, or by recruiting a larger sample of teachers with a highly integrated mixed-methods design with pre-observation and post-observation interviews. Also, other content aspects of the reading curriculum could be examined, or the instructional approaches in textbooks (e.g., differentiation, feedback practices), and how teachers deal with different approaches such as reciprocal teaching in small-groups (Okkinga et al., 2018). Also, it might be worthwhile to focus on the moderating impact of teachers’ own overall knowledge and beliefs with regards to reading comprehension in relation to their classroom practice and textbook use. This might give more insight into what is needed to have knowledgeable and flexible teachers who can compensate for sometimes less optimal materials. After all, reading comprehension is a complex skill to master, and probably even a more complex skill to teach.

## Supplementary Information

Below is the link to the electronic supplementary material.

Supplementary file1 (DOCX 32 kb)

## Footnotes

1 Please contact the first author for a matrix with a detailed overview of the units of analysis of the materials analysis, lesson observations, and the precise interview questions.
