# Supplementary material for: What textbooks offer and what teachers teach: an analysis of the Dutch reading comprehension curriculum
Source: Read Writ. 2022 Jan 6;35(7):1497–523. doi: 10.1007/s11145-021-10244-4 (PMC8734545; doi:10.1007/s11145-021-10244-4)
Supplement: Supplementary file 1 — Supplementary file1 (DOCX 32 kb) [file 11145_2021_10244_MOESM1_ESM.docx]

|  | **Materials Analysis** | **Interviews** | **Observations** |
| --- | --- | --- | --- |
| **Curricular structure & Objectives** | *Curricular structure*   - Number of lessons per year - Concentric or linear structure? - Availability of tests and monitoring? - Availability of preteaching modules? - Complete curriculum or teacher involvement needed (e.g., text selection)?   *Central objectives (listed by textbook) focus on:*   - Reading strategies - Genre knowledge / Text structures - Vocabulary growth - Other | *Curricular structure*   - How many lessons per week? How many minutes per lesson? - What is the curricular structure of the materials that you are using? How important do you believe this order/structure is? Do you change this order/structure in your teaching? - To what extent do the teaching materials help you to monitor student achievements? - Do you make use of supplementary materials? Which ones? Why?   *Central objectives*   - Why did you(r school) select this textbook? - What are the main objectives of the textbook, according to you? - Which aspects of it do you skip or put extra emphasis on, and why? | *Not applicable (as only one lesson is observed):*   - Write down: chapter, lesson; - Duration of lesson;   *Check if applicable:*   - Teacher makes connection to previous or next lesson; |
| **Lesson goals** | - Single lesson goal or multiple lesson goals? - Declarative knowledge focus (e.g., *After this lesson, you know the characteristics of a recipe*) - Procedural knowledge focus on ‘how to’ or activities (e.g. *After this lesson, you are able to use a dictionary*) - Conditional focus on when and why (*e.g. After this lesson, you know when and why it is best to use a Venn diagram to summarize a text*).   *If lesson goal is focused on reading strategy, then:*   - Strategies are related to phase: before (e.g., predicting, activating prior knowledge) / during (e.g., rereading, self-questioning) / after reading (e.g., summarizing, evaluating)   *Source*   - Lesson goals mentioned in manual / student materials   *Alignment*   - Number of exercises (un)related to lesson goal | - How do you use the lesson goals that are provided (e.g., mentioning it to students, using it to evaluate lesson, …)? Are you satisfied with the lesson goals? - If your textbook provides lessons with multiple lesson goals: do you teach the (extra) lessons in which students have to work with multiple reading strategies? How satisfied are you with these lessons? - How do you monitor whether your students have actually learnt something? Are you satisfied with this? - Do you adapt the texts (how and why)? To what extent do the texts help you to attain the prescribed lesson goals? | - Teacher tells students about lesson goals (write down if other lesson goal is provided than intended by textbook); - Teacher tells when/why attainment of lesson goal is important; - Teacher activates prior knowledge related to *lesson goal* (i.e., not activating knowledge about text topic, but about the main reading-related lesson goal)*;* |
| **Theory & instruction on strategies and text structures** | - Theory is provided written / orally and can be found in: teacher manual / student materials at the start / end / throughout lesson - Theory is focused on reading strategies / text topic or vocabulary / text structure or genre / other; - Theory focuses on genre (i.e. explaining the purpose and structural characteristics of a specific genre, such as a recipe or an interview); - Theory focuses on local text structure (i.e., explaining topics related to sentence-level structure, such as referential coherence or signaling words); - Theory focuses on global structure (i.e., topics that go beyond sentence-level structure, such as describing informational structures, story grammar, introduction-body-conclusion, or structural features of text lay-out (subdivision in paragraphs, (sub)headings)) that are different from simply explaining genres.   Specify: ___, ___, ___ (write down all key terms from theory related to reading strategies and text structures)   - Theory focuses on declarative knowledge aspects of strategy use or text structure knowledge (‘what’) - Theory focuses on declarative knowledge aspects of strategy use or text structure knowledge (‘how to’) - Theory focuses on declarative knowledge aspects of strategy use or text structure knowledge (‘when, why’, planning and evaluating)   *Mode of prescribed instruction*   - Teacher-led instruction: top-down, one-way flow of information from teacher to student; - Interactive discussion: a two-way flow of information from teacher to student; teacher uses questioning to expand students’ knowledge; - Teacher modeling: teacher models a reading strategy or how to cope with different text structures by thinking out loud, while students listen and observe. | - Can you carefully describe the different instructional activities in an average reading comprehension lesson? - What kind of things do you tell, explain, or demonstrate during instruction? - Do you demonstrate reading strategies? How? To what extent do the teaching materials help you to provide this instruction? Are you satisfied with this? - Do you tell students about the structure of texts? How? To what extent do the teaching materials help you to provide this instruction? Are you satisfied with this? | *Presence, order, and duration of instructional activities:*   - Teacher-led or interactive instruction __ mins - Modeling __ mins (+/- Reflection) - Instruction is focused on reading strategies / text topic or vocabulary / text structure or genre   Specify: ___, ____ ___ (write down all key terms from instruction)  *In case of modeling, check:*   - Teacher announces that he/she will model the strategy; - Teacher gives a concrete task for observation: ________________ - Teacher focuses modeling on text topic or vocabulary: _______ - Teacher focuses modeling on text structure: __________ - Teacher focuses modeling on reading strategy (without connection to text structure) _________ - Teacher makes explicit connections to instruction / lesson goal during/after modeling - Teacher models by thinking out loud as if there were no audience (I-pronoun) - Teacher is not actively involving students by asking interactive question - Teacher provides active reflection by discussing what was demonstrated during modeling - Teacher pays attention to students’ responses and provides feedback |
| **Student activities** | *Exercises*   - Textbook provides ‘text with questions’ as main activity - Number of exercises/questions per lesson - Number of exercises/questions related to lesson goal - Number of exercises/questions related to text structure - Number of exercises/questions related to planning and evaluating strategy use - Number of exercises/questions related to transfer   *Mode*   - Guided practice: students perform reading tasks under close supervision of their teacher who provides feedback and extra explanation where needed. - Collaborative learning: students perform reading tasks pair wise or in small groups - Individual practice: Students perform reading tasks independently on their own | - Can you carefully describe the different student activities in an average reading comprehension lesson? - Do you provide room for collaborative activities and independent practice? How? To what extent do your teaching materials help you to facilitate this? - How do you monitor whether your students have actually learnt something? To what extent do your teaching materials support you with this? | *Presence, order, and duration of instructional activities:*   - Guided practice: __ mins - Collaborative learning: __ mins - Individual practice: __ mins   *In case of guided practice, check:*   - Differentiation: prolonged instruction only for small group of students - Teacher asks student to read (part of) the text out loud to class - Teacher asks question(s) to the whole class and/or answers questions together with students - Teacher asks students to think out loud while applying strategies (peer modeling) - Teacher provides process / product feedback   *In case of collaborative learning, check:*   - Activity takes place in small groups or pairs (of ____ students) - Teacher makes use of specific collaborative learning structure and/or assigns different roles or tasks to students within groups (specify: ___________) - Teacher provides product / process feedback during / after activity   *Teacher assigns individual assignments*  *Students work individually* |
| **Transfer and metacognitive skills** | *Attention to transfer and metacognitive regulation*   - Domain: content-area subjects; writing/speaking; arts/creativity   *Type of transfer:*   - Text: lesson is focused on a text directly taken from a (social) sciences book or requires students to use a (social) sciences text to practice reading strategies and/or learn about text structure; - Suggestion: teacher manual provides open suggestions to practice strategies in the context of another domain; - Mandatory task: there are obligatory tasks/questions that explicitly require students to practice reading strategies in the context of another domain; - Optional task: ‘extra’ or ‘free choice’ tasks that explicitly require students to practice reading strategies in the context of another domain (see ‘Domain’). - Number of transfer tasks/exercises - Number of exercises/questions related to planning and evaluating strategy use | - How do you promote transfer? Do you try to integrate reading comprehension instruction with other subjects and skills? Can you give an example? - Do these teaching materials help you to integrate reading comprehension instruction with other subjects and/or skills? Why? - Do you also select your own texts? How and why? - Do you teach lessons in which students have to apply multiple reading strategies more independently (i.e., planning and evaluating)? How and why? | Check if applicable:   - Teacher selects lesson from regular textbook - Teacher makes explicit connection to another subject: specify: ___; - Teacher tells when/why attainment of lesson goal is important outside reading comprehension lesson; |
